# Inferring Foraging Areas of Nesting Loggerhead Turtles Using Satellite Telemetry and Stable Isotopes

**DOI:** 10.1371/journal.pone.0045335

**Published:** 2012-09-20

**Authors:** Simona A. Ceriani, James D. Roth, Daniel R. Evans, John F. Weishampel, Llewellyn M. Ehrhart

**Affiliations:** 1 Marine Turtle Research Group and Department of Biological Sciences, University of Central Florida, Orlando, Florida, United States of America; 2 Department of Biological Sciences, University of Manitoba, Winnipeg, Manitoba, Canada; 3 Sea Turtle Conservancy, Gainesville, Florida, United States of America; Monash University, Australia

## Abstract

In recent years, the use of intrinsic markers such as stable isotopes to link breeding and foraging grounds of migratory species has increased. Nevertheless, several assumptions still must be tested to interpret isotopic patterns found in the marine realm. We used a combination of satellite telemetry and stable isotope analysis to (i) identify key foraging grounds used by female loggerheads nesting in Florida and (ii) examine the relationship between stable isotope ratios and post-nesting migration destinations. We collected tissue samples for stable isotope analysis from 14 females equipped with satellite tags and an additional 57 untracked nesting females. Telemetry identified three post-nesting migratory pathways and associated non-breeding foraging grounds: (1) a seasonal continental shelf–constrained migratory pattern along the northeast U.S. coastline, (2) a non-breeding residency in southern foraging areas and (3) a residency in the waters adjacent to the breeding area. Isotopic variability in both δ^13^C and δ^15^N among individuals allowed identification of three distinct foraging aggregations. We used discriminant function analysis to examine how well δ^13^C and δ^15^N predict female post-nesting migration destination. The discriminant analysis classified correctly the foraging ground used for all but one individual and was used to predict putative feeding areas of untracked turtles. We provide the first documentation that the continental shelf of the Mid- and South Atlantic Bights are prime foraging areas for a large number (61%) of adult female loggerheads from the largest loggerhead nesting population in the western hemisphere and the second largest in the world. Our findings offer insights for future management efforts and suggest that this technique can be used to infer foraging strategies and residence areas *in lieu* of more expensive satellite telemetry, enabling sample sizes that are more representative at the population level.

## Introduction

The movement of organisms in space and time defines their interaction with the environment and, thus, constitutes a central aspect of their ecology and evolutionary biology [1]. How, where, and when organisms move also defines the array of resources they encounter, the range of threats they experience (predators, environmental conditions, anthropogenic hazards), and the degree to which they interact with other organisms. Migration, the regular seasonal movement of individuals, often from a breeding location to a nonbreeding location and back [2], is widespread in nature. Many species travel across thousands of kilometers in regular movements that constitute some of the most spectacular natural phenomena on the planet (e.g. Arctic tern [3], monarch butterfly [4], salmon [5], sea turtles [6], humpback whales [7]). Migratory connectivity describes the movement of individuals between breeding and nonbreeding areas. For many species the latter areas have not been identified [2].

Conserving migratory species has become a profound issue in the twenty-first century as habitats worldwide are being reduced in size or quality [1] (e.g. Nearctic migrant birds [8], Golden-cheeked Warbler [9], songbirds [10], monarch butterfly [11], salmon [12]). Thus, it is crucial to understand key migratory linkages in order to develop appropriate management and conservation measures in a rapidly changing world.

Our understanding of the ecology and evolution of migrating organisms has been impeded by the inability to observe directly their long distant movements. However, recent advances in satellite telemetry, genetic analysis and stable isotope analysis are unraveling geographical origin, movement patterns and foraging behavior of individual organisms. Until recently, tracking migratory animals involved the use of passive extrinsic markers (e.g. banding, patagial tags, numbered neck collars, streamers, flipper tags). In the last decade, stable isotope ratios have been increasingly used as intrinsic markers to trace foraging habits and movements of wildlife populations. Individuals that use geochemically different habitats, or feed on different resources, can be differentiated through use of stable isotope analysis because the isotopic profile of consumers reflects that of their prey in a predictable manner [13]. Consumers are typically enriched in δ^15^N relative to their food and, consequently, δ^15^N measurements serve as indicators of a consumer’s trophic position (given knowledge of prey species’ or baseline δ^15^N values), while δ^13^C values vary little along the food chain and are mainly used to identify location [14], [15], [16]. Moreover, the timescale over which dietary information is represented by stable isotope ratios (i.e., residence time) varies with tissue type and depends largely upon metabolic turnover [17].

Isotopic signatures may be influenced by diet, habitat type and geographic location. Differences among and within oceanic regions in nutrient cycling at the base of the food web produce geographical gradients in carbon and nitrogen isotope composition [13]. For example, both carbon and nitrogen stable isotope ratios can provide information on foraging latitude because phytoplankton have higher δ^13^C and lower δ^15^N values in temperate than in higher-latitude ecosystems [13], [18]. Despite the widespread use of this technique in marine systems, geographic variation in stable isotope ratios at the base of the food web have been described only at very coarse scales [13]. Few regional maps of marine isoscapes (spatially explicit regions of stable isotope ratios) are available, thereby limiting the use of isotopic methods in the marine realm. However, another way to interpret the carbon signature of top predators is to calibrate isoscapes using top predators themselves (Pacific humpback whales [7], Pacific bigeye and yellowfin tuna [13], albatrosses [19]).

Loggerhead turtles (*Caretta caretta,* L.) are highly migratory organisms with a complex life cycle. Loggerheads exhibit weak connectivity (*sensu* Webster [2]); that is, individuals at a breeding area may travel to different foraging grounds and individuals at a foraging ground may return to different breeding areas. Only some key foraging grounds have been identified so far using satellite telemetry. In the last decade, stable isotope analysis and satellite tracking have provided insight into loggerhead feeding ecology and migration. Hatase et al. [20] demonstrated that some adult female loggerheads nesting in Japan inhabit oceanic zones rather than neritic habitats, which differs from the accepted life-history model for this species [21]. Likewise, McClellan and Read [22], [23] described a behavioral dichotomy among immature loggerheads that alternate between neritic and oceanic habitat. More recently, Zbinden et al. [24] used a combination of satellite telemetry and stable isotope analysis to assign foraging areas of untracked loggerheads nesting in Greece, and Pajuelo et al. [25] used a combination of the two techniques to investigate post-mating destinations of male loggerheads from a breeding aggregation in Florida. Using stable isotope analysis and epibionts from loggerheads nesting on the east coast of Florida, Reich et al. [26] found a bimodal distribution of δ^13^C that could reflect a bimodal foraging strategy that the authors interpreted as a nearshore/offshore dichotomy or–because of the potential for confusion among four gradients of δ^13^C in marine environments - a polymodal foraging strategy. Reich et al. [26] called for integrated studies in which sufficient numbers of individuals are fitted with satellite transmitters and passive tags and are sampled for stable isotope analysis, epibionts and other biomarkers to evaluate further the foraging strategies and foraging habitats of Florida loggerheads. While there has been extensive tracking effort on loggerheads nesting along the Florida west coast [27], [28] (Tucker unpublished), a paucity of tracking studies have focused on loggerhead nesting on the Florida east coast, despite the fact that the latter accounts for approximately 80% of all the nesting activity in the United States [29]. Furthermore, few studies have measured stable isotope ratios in marine megafauna in the western North Atlantic (sharks [30], Atlantic Bluefin tuna [31], leatherback turtles [32], loggerheads [25]).

In this study using a combination of satellite telemetry and stable isotope analysis, we (1) identified key foraging grounds used by female loggerheads nesting in Florida and (2) examined the relationship between stable isotope ratios and the location of nonbreeding foraging areas. This is the first study integrating satellite telemetry and stable isotope analysis to investigate migratory strategies used by loggerhead females in the Atlantic Ocean. If loggerhead isotopic signatures from distinct foraging areas differ significantly, stable isotope analysis may be considered a viable alternative to satellite telemetry for denoting migratory patterns in the NW Atlantic, as found elsewhere [33], [34]. Knowledge of foraging grounds and migratory connectivity for loggerheads in the NW Atlantic is crucial to develop appropriate conservation measures and help managers define and protect loggerhead critical habitat.

## Methods

### Ethics Statement

The animal use protocol for this research was reviewed and approved by the University of Central Florida Institutional Animal Care and Use Committee (IACUC protocol #09–22W). Procedures were approved under the Florida Fish and Wildlife Conservation Commission (Marine Turtle Permit #025).

### Biology and Conservation Status of Loggerhead Turtles

Loggerheads are highly migratory organisms with a complex life cycle where different life stages occupy different ecological environments. They typically switch from an initial oceanic juvenile stage to one in the neritic zone, where maturity is reached. Breeding migrations are subsequently undertaken every two to three years [21]. Loggerheads are largely carnivorous during all life history stages [35], [36]. The loggerhead turtle is classified as endangered by the IUCN Red List [37] and listed as 9 distinct population segments (4 of which are threatened and 5 endangered) under the U.S. Endangered Species Act [38] (2011). The Northwest Atlantic Ocean distinct population segment is classified as threatened under the U.S. Endangered Species Act. In 2008, the U.S. National Marine Fisheries Service (NMFS) and the U.S. Fish & Wildlife Service issued a second revision of the North West Atlantic (NWA) loggerhead recovery plan. Five Recovery Units (management subunits of a listed species that are geographically or otherwise identifiable and essential to the recovery of the species) have been identified based on genetic differences and a combination of geographic distribution of nesting densities and geographic separation [39]. The NWA Peninsular Florida Recovery Unit, which comprises loggerheads nesting from the Florida/Georgia border through Pinellas County (Florida), is the largest loggerhead nesting population in the western hemisphere and one of the two largest in the world [29]. Florida’s long-term loggerhead nesting trend indicates a nesting decline of 16% from 1998 to 2011 [40] but the reasons for the observed decline in nest numbers are unclear [41]. In a recent analysis of nesting trends in Florida, Witherington et al. [42] argued that the reduction in annual nest numbers could be best explained by a decline in the number of adult female loggerheads in the population. Although multiple stressors are likely responsible for the decline in adult females, fishery by-catch ranked first in the analysis of threat factors for adult females [42] and has been identified as a major threat for the recovery of the Northwest Atlantic loggerhead population [43]. Only some key foraging grounds for the NWA Florida Peninsular Recovery Unit population have been identified so far using satellite telemetry: the Bahamas, Cuba, the West Coast of Florida, the Yucatán Peninsula of Mexico and the Gulf of Mexico [27], [28], [44]. A recent paper on the global priorities for sea turtle conservation in the 21^st^ Century highlights the need to identify key foraging grounds and oceanic hotspots to develop informed management plans for the recovery of the species [45].

### Study Site and Sampling

Blood samples were collected for stable isotope analysis from turtles nesting within the 21 km stretch of beach of the Archie Carr National Wildlife Refuge (hereafter Carr NWR) located in southern Brevard County on Florida’s east-central coast. This area hosts the most important loggerhead rookery in the western hemisphere and accounts for approximately 25% of all the loggerhead nests in Florida [29]. Here, all nesting activity is monitored and a subsample of females is encountered and tagged using both Inconel flipper tags and passive integrated transponders during night surveys. A total of 71 females, 14 of which were equipped with a satellite tag, were included in this study.

### Tracking Analysis

Between 2008 and 2010, we attached satellite transmitters (Wildlife Computers MK10-A and MK10 AFB, Redmond, Washington, USA and SIRTRACK KiwiSat 101 K1G 291A, New Zealand) to 14 female loggerheads and tracked their post-nesting migration (Table 1). Half of the units were deployed at the beginning of the nesting season on turtles previously marked (with Inconel flipper tags) as part of a different project investigating clutch frequency, movements and foraging activity during the inter-nesting period. The remaining seven tags were deployed at the end of July of each year in collaboration with the Sea Turtle Conservancy, a Florida based non-profit organization. Transmitters were affixed to the turtle’s carapace (between the first and second vertebral scute) using two cool-setting two-part epoxies (Power Fast and Sonic Weld). Females were kept in a wooden box during attachment and released at the capture location a few hours later. Satellite tags were programmed to transmit daily over a 24 h period during the nesting season (beginning of May to end of August) and every other day outside of the nesting season to extend battery life. Service Argos, Inc provided position estimates and associated location accuracy. To reject implausible locations, we employed a customized script in the R package software that was based on a two-stage filtering algorithm (land/sea and Freitas’ speed-distance-angle filters [46]). Sea turtle movements were reconstructed by plotting the best location estimate per day of the filtered location data using ArcGIS version 10.0. If two or more high quality locations were received, we only used the first received for that day. Migratory destination was classified as ‘oceanic’ if a turtle moved off the continental shelf, as defined by the 200 m isobath, or ‘neritic’ if it remained on the shelf.

**Table 1:** Information on satellite tracking and foraging area of choice of 14 satellite-tracked loggerheads.

Turtle ID	PTT eployment date	Trackingduration (d)	Date of lastlocation	Foraging area	PTT type
A	31 July 2008	1397	28 May 2012	North (MAB)	KiwiSat 101
B	05 May 2009	873	30 Sept 2011	North (MAB)	Mk10-AFB
c	12 May 2009	530	21 Oct 2010	North (MAB)	Mk10-AFB
d	19 May 2010	188	23 Nov 2010	North (MAB)	Mk10-A
e	19 May 2010	286	1 March 2011	North (MAB)	Mk10-A
f	20 May 2010	380	4 June 2011	North (MAB)	Mk10-A
g	1 Aug 2009	60	30 Sept 2009	Central (SAB)	KiwiSat 101
h	1 Aug 2010	204	21 Feb 2011	Central (SAB)	Mk10-A
i	31 Jul 2010	127	7 Dec 2010	Central (SAB)	Mk10-A
j	31 Jul 2010	90	29 Oct 2010	Central (SAB)	KiwiSat 101
k	31 Jul 2008	795	16 Feb 2011	South (SE GoM)	KiwiSat 101
l	21 May 2009	932	9 Dec 2011	South (Bahamas)	Mk10-AFB
m	29 May 2009	478	19 Sept 2010	South (FL Keys)	Mk10-AFB
n	30 July 2009	378	12 Aug 2010	South (Bahamas)	KiwiSat 101

Abbreviations are as follow: platform terminal transmitter (PTT), day (d), Mid-Atlantic Bight (MAB), South-Atlantic Bight (SAB), South East Gulf of Mexico (SE GoM), Florida Keys (FL Keys).

**Figure 1:**
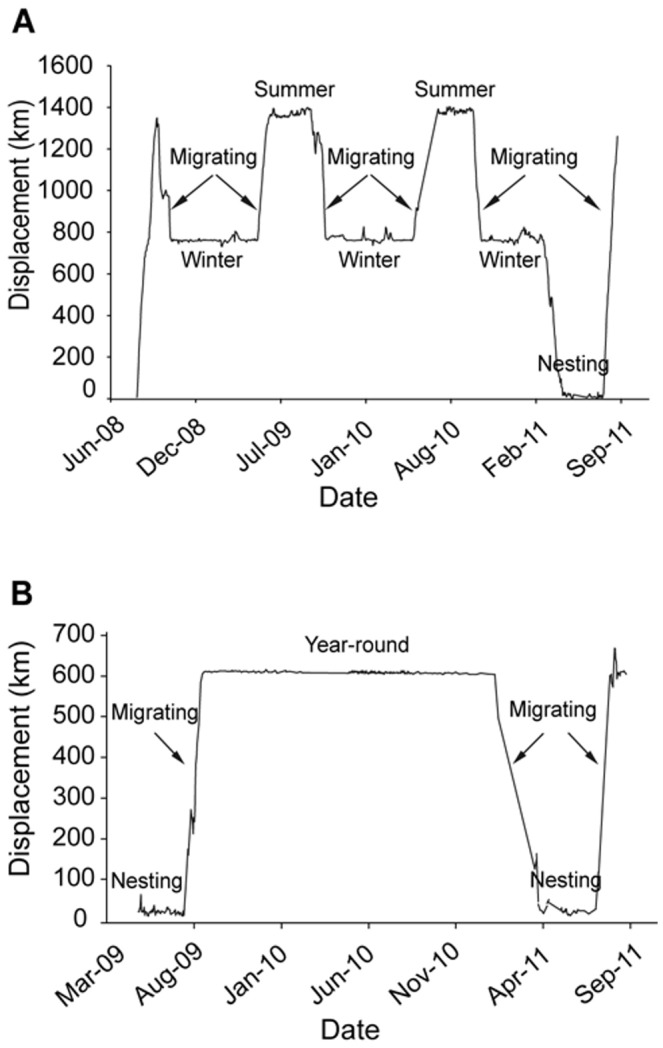
Displacement from release site plot of loggerheads equipped with satellite tags. (A) Displacement pattern of a turtle that followed the northern strategy and migrated between summer and winter foraging areas (turtle a, see Table 1 for details). Females following the northern strategy moved between summer foraging grounds in the Mid-Atlantic Bight (MAB) off the Delmarva Peninsula and winter foraging grounds located in the waters off North Carolina. (B) Displacement pattern of a turtle that took up year-round residence in the Great Bahamas Bank and did not show seasonal migration (turtle l). Phases of migration are represented by rapid changes in displacement distance; summer, winter and year-round foraging areas can be seen where displacement values plateau.

To investigate the relationship between foraging areas identified by telemetry and isotopic signatures of female tissues, we calculated average latitude and longitude of foraging grounds. We define foraging ground as the area where an individual loggerhead resides during the nonbreeding season and migration as the movement between foraging areas (if more than one foraging area is used, Figure 1A) or between foraging area and nesting area (Figure 1A, B). Migration, summer and winter foraging phases were determined by plotting displacement from deployment site (Figure 1). Migration was considered to have ended when displacement began to plateau. Likewise summer and winter foraging phases were considered to have ceased when displacement values started to change again [47]. To calculate mean latitudes and longitudes of summer and winter foraging areas, we averaged the locations of all filtered data (best estimate/day) from each plateau. If a tag transmitted for more than one year and the individual made multiple seasonal movements (Figure 1A: winter 2009-summer 2009-winter 2010-summer 2010-winter 2011), we averaged all filtered data from the summer plateaus (summer 2009 and 2010) and the winter plateaus (winter 2009, 2010, 2011) in order to obtain a unique latitude and longitude value representing the overall turtle summer and winter foraging area. We then used mean latitude and longitude to calculate the distance to the nearest coastline (distance from shore, km).

### Stable Isotope Sampling and Analysis

Blood samples (4 ml) were collected from the cervical sinus with a 20-gauge needle and syringe [48] as soon as the turtle began to cover her nest. Blood was transferred to a non-heparanized container and separated into serum and cellular components by centrifugation (5000 rpm×10 min), then frozen at −20°C until analysis. To address our objectives, we measured the stable isotope ratios of red blood cells (RBC), a tissue assumed to have a long turnover rate that should reflect an integration of diet and habitat at the foraging ground prior to breeding migration. Tissue turnover rate for RBCs in adult sea turtles is unknown but it has been estimated to reflect the foraging habits of the 4–7 months prior sampling [49], [50] (Ceriani et al. unpublished). We assumed females exhibit site fidelity to foraging grounds (pre-nesting foraging area  =  post-nesting foraging area). This assumption is commonly used in studies combining telemetry and stable isotope analysis [20], [24], [51], [52] and is supported by the data available for individual marine turtles that have been equipped repeatedly with satellite tags [47], [53] and by long-term studies at foraging grounds [54]. Recently, site fidelity in female loggerheads has been indicated by the long-term consistency in isotopic signatures of scute layers, a tissue that incorporates several years of dietary history and habitat use [55]. Moreover, if our analysis finds concordance among individual turtleδ^13^C and δ^15^N groupings and distinct post-nesting migratory destinations, our study will provide further evidence supporting foraging ground philopatry in most adult loggerhead females.

Sample preparation was done at the Biology Department of the University of Central Florida. Samples were prepared following standard procedure. RBC samples were freeze-dried for 48 h before being homogenized with mortar and pestle. Lipids were removed using a Soxhlet apparatus with petroleum ether as solvent for 12 h. Approximately 0.5 mg of each sample was weighed and sealed in tin capsules. Prepared samples were sent to the Stable Isotope Core Laboratory at Washington State University, where they were converted to N_2_ and CO_2_ with an elemental analyzer (ECS 4010, Costech Analytical, Valencia, CA) and analyzed with a continuous flow isotope ratio mass spectrometer (Delta PlusXP, Thermofinnigan, Bremen). Isotopic reference materials were interspersed with samples for calibration. Stable isotope ratios were expressed in conventional notation as parts per thousand (‰) according to the following equation:
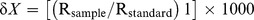
where *X* is ^15^N or ^13^C, and R is the corresponding ratio ^15^N:^14^N or ^13^C:^12^C. The standards used for ^15^N and ^13^C were atmospheric nitrogen and Peedee Belemnite, respectively. Precision was 0.07‰ for δ^13^C measurements and 0.11‰ for δ^15^N.

### Statistical Analysis

Relationships between δ^13^C and δ^15^N and mean latitude of foraging ground and distance from shore were explored through multiple regression analysis. Akaike’s Information Criteria (AIC) was used to determine the best fitting regression [56]. We included distance from shore in the multiple regression analysis to take into account differences in coastline shape and female differential use of the continental shelf (inner, mid or outer shelf). Because some females undertook a seasonal migration and it is unknown whether RBC isotopic signatures reflect the diet and geographic location occupied during the summer or winter months, we performed two distinct multiple regression analyses. In one we used mean latitude and distance from shore of summer areas identified from telemetry, while in the other we used mean latitude and distance from shore of winter areas. The remaining females did not exhibit a seasonal migration and, therefore, we calculated only one average latitude and distance from shore.

To test for significant differences in isotopic signatures among foraging areas, we used multivariate analysis of variance (MANOVA) with the Pillai’s trace test. Data were tested for normality and homogeneity of variance using Kolmogorov-Smirnov and Levene’s test, respectively. Data were normal but did not meet the equal variance assumption even after transformation. We chose the Pillai’s trace test because it is the most robust of the tests when the assumption of similar-covariance matrix is not met [57]. We used post hoc Games-Howell (GH) multiple comparison tests (which assumes unequal variance) to identify groups responsible for statistical differences [58]. We used discriminant function analysis (DFA) to examine how well δ^13^C and δ^15^N predict the post-nesting foraging grounds used by loggerheads. We used δ^13^C and δ^15^N values of the 14 females equipped with satellite tags as training data set (with equal priors for the classification) to develop the discriminant functions and the untracked turtles as test data set for the discriminant classification. Untracked turtles are defined as females that were sampled for stable isotope analysis but that were not equipped with satellite tags. Data were analyzed using program R (R Development Core Team 2009), SPSS v. 19, Sigma Plot 10.0 and ArcGIS 10.0. Alpha level was set to 0.05 for all statistical analyses.

## Results

### Satellite Telemetry & Post-nesting Migration Destinations

**Figure 2:**
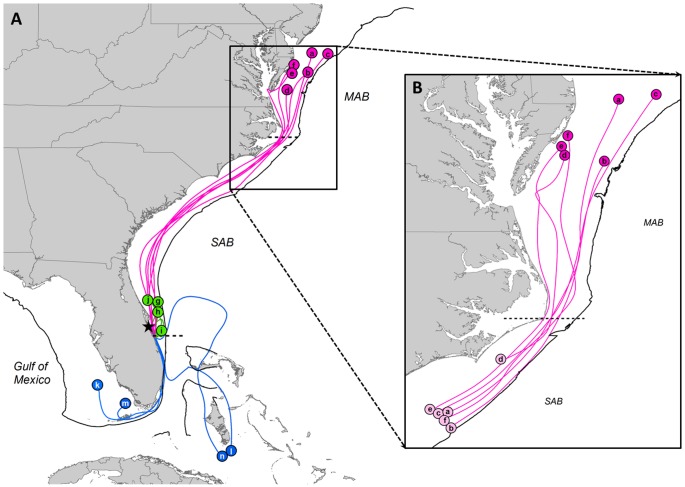
Reconstructed satellite tracks (n = 14) of loggerheads tagged after nesting at the Carr NWR. (A) Reconstructed route (pink, green and blue lines) to foraging areas (labeled circles) for individuals *a* to *n* from release location (black star). Loggerheads were classified into three migratory groups: northern (a to f), central Florida resident (g to j) and southern (k to n). Pink, green and blue reconstructed routes represent northern, resident and southern migratory groups, respectively. (B) Reconstructed route (pink lines) from summer foraging areas (darker pink-labeled circles) to wintering areas (lighter pink-labeled circles) for individuals that followed the northern strategy (a to f). The 200 m isobath is delineated (black line). Dotted line separates Mid-Atlantic Bight (MAB) and South-Atlantic Bight (SAB). A bight is defined as a long, gradual bend or recess in the coastline that forms a large, open bay. The MAB is defined as the region enclosed by the coastline from Cape Cod (MA), to Cape Hatteras (NC). The SAB extends from Cape Hatteras (NC) to West Palm Beach (FL).

Loggerheads moved across a wide range of latitudes spanning from the Great Bahamas Bank (23°N) to the offshore waters of Virginia and Delaware (38.6°N). Satellite telemetry identified three migratory pathways and associated foraging grounds (Figure 2): (1) a seasonal shelf-constrained North-South migratory pattern between waters offshore Virginia/Delaware and North Carolina (along the NE USA coastline), (2) a year-round residency in southern foraging grounds (Bahamas and SE Gulf of Mexico) and (3) a residency in the waters adjacent to the breeding area (eastern central Florida). We classified female loggerheads into three migratory strategies according to whether they migrated “north” (northern), “south” (southern) or stayed in central Florida (resident or central) and will follow this classification hereafter. Migratory destinations of the 14 females were classified as “neritic” since all individuals took up residency within the limits of the continental shelf (water depth <200 m).

At the end of the nesting season, six individuals departed eastern central Florida and migrated north to seasonal foraging grounds above 35°N in the Mid-Atlantic Bight where they spent the rest of the summer and beginning of fall (Figure 2A). By the end of October, these six individuals left summer feeding areas and migrated south toward winter grounds located in North Carolina between Cape Hatteras and Wilmington where they stayed until the beginning of May (Figure 2B). Three of these six females, whose tracking lasted more than 1 year, exhibited the same seasonal displacement among years (Figure 1A, Figure S1). Four females that were equipped with tags at the end of the nesting season (Table 1, individuals g-j) did not leave the area of eastern central Florida but remained in the waters off Cape Canaveral (Figure 2A, Figure S2). Tracking data for these 4 individuals were limited since tags failed between 2 and 7 months from deployment. However, females that undertook long-distance post-nesting migrations (all but individuals g-j in Table 1) left the breeding area by mid-August, immediately after laying the last nest of the season, and traveled a minimum of 288 km during the first two months after deployment (northern: 1205 km ±121 km; southern: 458 km ±171 km). Therefore, since these 4 loggerheads did not lay additional clutches and did not depart from the area (displacement after 2 months at large: 89 km ±52 km), we assumed eastern central Florida to be their final destination. The remaining 4 females headed to subtropical northwest Atlantic and southeast Gulf of Mexico foraging areas where they remained year-round until the next breeding migration (Figure 1B, Figure 2A, Figure S3). Two females took up year-round residency in the Great Bahamas Bank, just south of the Bahamian island of Andros, one female dwelled in the shallow waters of the Gulf of Mexico immediately west of the Florida Keys, while the last individual resided in the SE Gulf of Mexico off the SW Florida coast. Even though loggerheads that migrated south used two geographic regions (the Bahamas Great Bank vs. the Gulf of Mexico) with distinctive oceanographic regimes, we refrained from splitting the southern aggregation due to the small sample size of loggerheads equipped with satellite tags.

### Geographic Variability in Stable Isotope Ratios

**Figure 3:**
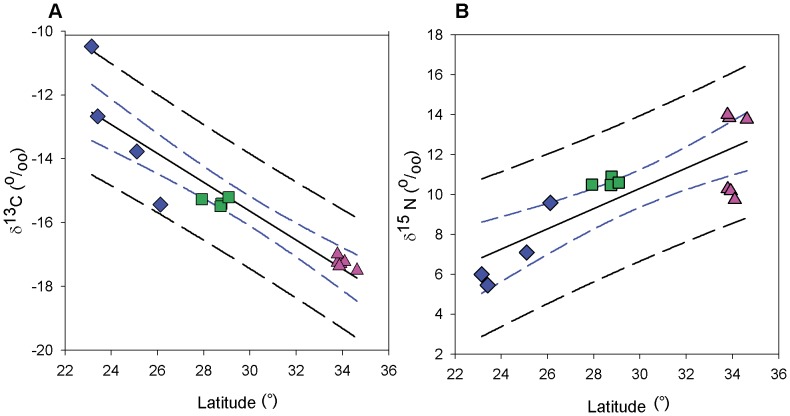
Relationship between RBC stable isotope ratios and post-nesting foraging ground location. RBC δ^13^C (A) and δ^15^N (B) values of satellite-tracked adult female loggerheads (n = 14) versus mean latitudes of winter foraging areas calculated based on satellite telemetry. Blue diamonds represent individuals migrating to southern foraging grounds (southern), green squares females residing in eastern central Florida (resident) and pink triangles females that migrated to northern foraging areas (northern). Only northern loggerheads undertook seasonal migration between winter and summer foraging ground. In the case of northern females, the latitude plotted represents the average latitude of the winter foraging area for each individual. The remaining eight females did not show seasonal movement; therefore, the latitude plotted represents the average latitude of the year-round foraging area. Dashed blue and black lines indicate 95% confidence and predictive interval (respectively) for the regression analysis.

The δ^13^C values of RBCs from tracked female loggerheads ranged from −17.50 ‰ to −10.48 ‰, and δ^15^N varied between 5.46 ‰ and 14.00 ‰. The multiple regression analysis and AIC model selection revealed that average latitude alone was the best predictor of δ^13^C values in female tissues for both winter (Table 2) and summer (Table 3) feeding areas. δ^13^C decreased significantly with increasing latitude for both winter feeding areas (F1,12 = 75.04, r^2^ = 0.862, p<0.001, Figure 3A) and summer feeding areas (F1,12 = 46.13, r^2^ = 0.794, p<0.001). Likewise, winter feeding area latitude was the best explanatory variable for δ^15^N (F1,12 = 23.01, r^2^ = 0.657, p<0.001; Figure 3B), while the additive model of latitude and distance from shore explained the relationship better than latitude alone with regard to summer feeding areas (F1,12 = 21.96, Adjusted r^2^ = 0.763, p<0.001).

**Table 2:** Comparison of linear regression models describing the relationship between RBC δ^13^C and δ^15^N and geographic location of winter non-breeding foraging areas for the 14 loggerheads fitted with satellite tags.

	Model variables	R^2^	Adj.R^2^	RSS	N	K	AIC_c_	Δ AICc	AICc Weights	P
**δ ^13^C**	**lat**	**0.862**	**0.851**	**0.797**	**14**	**3**	**−31.7**	**0**	**0.885**	**<0.0001**
	lat + dist shore	0.870	0.846	0.808	14	4	**−**27.5	4.2	0.106	
	lat * dist shore	0.877	0.840	0.825	14	5	**−**22.1	9.6	0.007	
	dist shore	0.121	0.048	2.013	14	3	**−**18.8	13.0	0.001	
**δ ^15^N**	**lat**	**0.657**	**0.629**	**1.617**	**14**	**3**	**−21.8**	**0.0**	**0.818**	**0.0004**
	lat + dist shore	0.714	0.662	1.543	14	4	**−**18.4	3.4	0.150	
	dist shore	0.026	**−**0.055	2.726	14	3	**−**14.5	7.3	0.021	
	lat * dist shore	0.732	0.652	1.566	14	5	**−**13.2	8.7	0.011	

Model selection used Akaike’s Information Criterion, corrected for small sample sizes (AIC_c_). Abbreviations are as follow: RSS  =  residual sum of squares, N  =  number of observations, K  =  number of parameters, ΔAIC_c_  =  difference between each model and the best model, AIC_c_ weight  =  relative information content, P  =  probability associated with the best model, lat  =  average latitude of foraging ground based on tracking data, dist shore  =  distance from shore (in km) calculated from the point having as coordinates average latitude and longitude of foraging ground, lat * dist shore  =  lat + dist shore + lat * dist shore.

**Table 3:** Comparison of linear regression models describing the relationship between RBC δ^13^C and δ^15^N and geographic location of summer non-breeding foraging areas for the 14 loggerheads fitted with satellite tags.

	Model variables	R^2^	Adj.R^2^	RSS	N	K	AIC_c_	Δ AICc	AICc Weights	P
**δ ^13^C**	**lat**	**0.794**	**0.776**	**0.976**	**14**	**3**	**−28.9**	**0**	**0.884**	**<0.0001**
	lat + dist shore	0.804	0.768	0.994	14	4	**−**24.6	4.3	0.103	
	lat * dist shore	0.826	0.774	0.981	14	5	**−**19.7	9.2	0.009	
	dist shore	0.026	−0.055	2.119	14	3	**−**18.0	10.9	0.004	
**δ ^15^N**	**lat + dist shore**	**0.800**	**0.763**	**1.291**	**14**	**4**	**−20.9**	**0.0**	**0.551**	**0.0001**
	lat	0.549	0.511	1.855	14	3	**−**19.9	1.0	0.329	
	dist shore	0.304	0.246	2.304	14	3	**−**16.9	4.1	0.072	
	lat * dist shore	0.823	0.769	1.275	14	5	**−**16.0	4.9	0.048	

Model selection used Akaike’s Information Criterion, corrected for small sample sizes (AIC_c_). Abbreviations are as follow: RSS  =  residual sum of squares, N  =  number of observations, K  =  number of parameters, ΔAIC_c_  =  difference between each model and the best model, AIC_c_ weight  =  relative information content, P  =  probability associated with the best model, lat  =  average latitude of foraging ground based on tracking data, dist shore  =  distance from shore (in km) calculated from the point having as coordinates average latitude and longitude of foraging ground, lat * dist shore  =  lat + dist shore + lat * dist shore.

**Figure 4:**
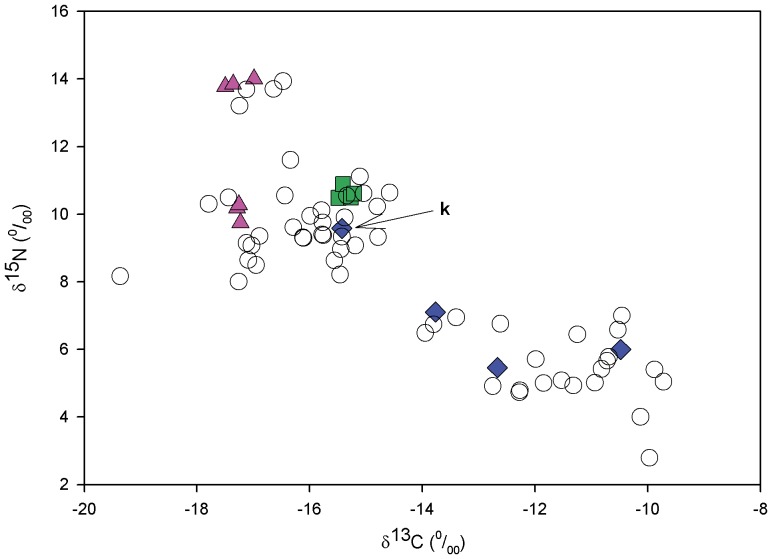
Scatterplot of δ^13^C and δ^15^N values for the 71 nesting loggerhead turtles sampled at the Carr NWR, Florida (USA). Pink triangles represent females equipped with satellite tags that migrated to northern foraging areas, green squares those foraging in eastern central Florida, blue diamonds those foraging in the south, while empty circles represent untracked females. The arrow indicates turtle “k”, which foraged in the SE Gulf of Mexico. The δ^13^C and δ^15^N values of this individual were extremely similar to the ones found in eastern central Florida residents, while the average latitude of the foraging ground used by this female for almost two years was intermediate between residents and the other southern individuals. RBC stable isotope ratios of untracked females (n = 57) have a similar distribution pattern to the 14 satellite-tracked loggerheads.

Females from the three foraging areas segregated by their overall isotopic signatures (MANOVA, Pillai’s trace test, F_4, 22_ = 4.147, p = 0.012) and, in univariate analysis, both δ^13^C (ANOVA, F_2, 11_ = 17.695, p<0.001) and δ^15^N values (F_2, 11_ = 10.217, p = 0.003) differed among foraging aggregations (Figure 4). Mean δ^13^C values per group varied from –17.27±0.17‰ in females using northern foraging areas to –13.09±2.08‰ in southern individuals. δ^15^N values ranged from 11.97±2.09‰ (northern females) to 7.04±1.83‰ (southern females). Individuals residing in eastern central Florida exhibited intermediate values between northern and southern loggerheads in both δ^13^C (−15.35±0.13‰) and δ^15^N (10.62±0.19‰). Post hoc Games-Howell (GH) multiple comparison tests indicated that the northern aggregation δ^13^C differed significantly from the resident aggregation (p<0.001) and marginally from the southern (p = 0.054), while resident and southern aggregations did not differ from each other in δ^13^C (p = 0.222). δ^15^N signatures of loggerheads using southern foraging areas differed significantly from the northern aggregation (p = 0.013) and marginally from the resident (p = 0.058) group, while northern and resident aggregations did not differ from each other in δ^15^N (p = 0.336).

### Assignment of Untracked Females to Foraging Areas

**Figure 5:**
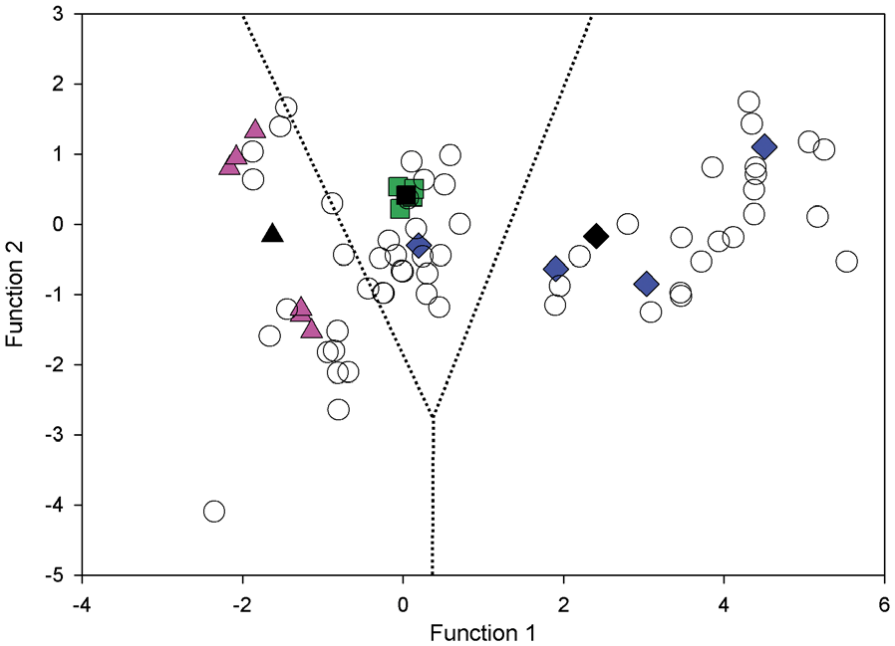
Discriminant function analysis (DFA) of foraging groups based on the stable isotope ratios. Function 1 accounted for 97.6% of the between-group variability. Pink triangles represent females equipped with satellite tags that migrated to northern foraging areas, green squares those foraging in eastern central Florida and blue diamonds those foraging in the south. Black markers represent the centroids for the respective foraging groups. Empty circles represent untracked females. Dotted lines define the three DFA territories.

The discriminant analysis of the training data set (14 loggerheads equipped with satellite tags) was significant (P> Wilks’ Lambda <0.002). Two discriminant functions were calculated, with a combined Χ^2^ (4) = 16.785, p = 0.002. After removal of the first function, the association between groups (foraging areas) and predictors (δ^13^C and δ^15^N) became not significant Χ^2^ (1) = 0.867, p = 0.352. The first discriminant function accounted for 97.6% of the between-group variability. Overall the discriminant analysis of the training data set was able to correctly classify the foraging ground used for all but one individual (92.9% of original grouped cases correctly classified). The only misclassified loggerhead was assigned to the resident aggregation, while satellite telemetry indicated this loggerhead belonged to the southern aggregation as it migrated to the SE Gulf of Mexico. The stability of the classification procedure was checked by a leave-one-out cross validation, which classified 92.9% of the test data set correctly. In the untracked females, RBC δ^13^C ranged from −19.36 ‰ to −9.72 ‰ and δ^15^N varied between 2.79 ‰ and 14.00 ‰. Putative foraging ground was predicted for 57 untracked turtles in the test data set and was based on the above classification functions. The discrimination analysis assigned 15 of the 57 untracked individuals (26.3%) to the northern aggregation, 20 females (35.1%) to the resident group and 22 females (38.6%) to the southern aggregation (Figure 5, Table 4). When we considered the entire dataset (n = 71), the relative importance of the three foraging areas remains similar with 21 females considered northern (29.6% of all females), 24 resident (33.8% of all females) and 26 southern (36.6% of all females).

**Table 4:** Foraging ground assignment (number and %) for the discriminant model based on δ^13^C and δ^15^N values of loggerhead RBCs.

		Predicted Group Membership			
Group		Northern	Central	Southern	Total
**Training data** **(n = 14)**	**Northern**	6 (100%)	0 (0%)	0 (0%0	6
	**Central**	0 (0%)	4 (100%)	0 (0%)	4
	**Southern**	0 (0%)	1 (25%)	3 (75%)	4
**Test data** **(n = 57)**	**Untracked**	15 (26.3%)	20 (35.1%)	22 (38.6%)	57
	**Total**	**21**	**25**	**25**	**71**

Number and % of loggerheads assigned to each foraging ground based on the classification results. Observed classes are in rows, predicted in columns. We used δ^13^C and δ^15^N values of the females equipped with satellite tags (n = 14) as training data set to develop the discriminant functions and the untracked turtles (n = 57) as test data set for the discriminant classification. 92.9% of original and of cross-validated grouped cases were classified correctly. Only one southern individual (turtle ID k, Table 1) was misclassified and assigned to the central group.

## Discussion

### Satellite Telemetry

Our telemetry data identified new foraging areas used by female loggerheads of the NWA Florida Peninsular Recovery Unit. Six of the 14 individuals we tracked moved north and four resided in eastern central Florida, demonstrating for the first time that the Mid- and South-Atlantic Bights, respectively, provide important foraging grounds for adult females of this Recovery Unit. This result is a major difference from the results of prior satellite tracking studies. Overall there are published tracking data for 47 females of the NWA Florida Peninsular Recovery Unit [27], [28], [44]. Prior to this study, only 19 females were tracked (between 1988 and 2000) from eastern central Florida [27], [44] despite the fact that the Carr NWR alone accounts for ∼25% of the 30–60,000 nests laid in Florida each year [29], [42]. Only one of the 19 previously tracked individuals moved north to North Carolina and one stayed in eastern central Florida, while the remaining 17 females migrated south along the east coast of Florida to the Bahamas Archipelago, Cuba, west coast of Florida and Gulf of Mexico.

The Mid- and South- Atlantic Bights are known to be important foraging areas for adult females of the NWA North Recovery Unit, which comprises loggerheads nesting from the Florida/Georgia border to southern Virginia [39]. Of the 73 females of the NWA North Recovery Unit equipped with satellite tags between 1997 and 2008 in North Carolina, South Carolina and Georgia, 51 used the north strategy, nine stayed year-round in the South Atlantic bight, four migrated to the Bahamas, Florida Keys and Gulf of Mexico, while the remaining ceased transmitting before reaching post-nesting migration destinations [47], [59], [60].

Prior to our study, the documentation that adult females of the NWA Florida Peninsular Recovery Unit used Mid- and South- Atlantic Bights were limited to few flipper tag returns [61]. In fact, the majority of tag returns for this Recovery Unit are from Cuba [62], Bahamas and Florida Keys (Ehrhart, unpublished). Interestingly, migratory patterns similar to the ones we identified have been shown recently in male loggerheads tracked from Cape Canaveral (FL, USA), a major breeding aggregation only 40 km north of our study site [63]. Twenty of the 29 males tracked used the Mid- (n = 8) and South Atlantic (n = 12) Bights. Among the 12 males that used the South Atlantic Bight, two individuals migrated to South Carolina, while 10 remained in eastern central Florida suggesting that eastern central Florida supports a year round aggregation of adult loggerheads.

We can think of three plausible explanations for the novelty of our tracking data: (1) the high use of Mid- and South- Atlantic Bights may be a new phenomenon, (2) sample size of telemetry studies is small and our results, as well as prior studies’, may be due to chance, (3) Mid- and South- Atlantic Bights have always been important foraging grounds for the Florida Peninsular Recovery Unit but the importance was not detected with prior technology such as flipper tag return. Even though considerable progress has been made into understanding sea turtle migration using recovery of flipper-tagged individuals [61], [62], [64], [65], [66], [67], [68], the use of this technique to assess post-nesting migration destinations has some drawbacks. Flipper tag recapture distribution may be affected by small sample sizes, differential fishing pressure and/or oceanographic features such as currents that may push carcasses offshore. In recent years advances in satellite telemetry, genetic analysis and stable isotope analysis have provided additional tools to unravel migratory connectivity. While it is not possible to discriminate between hypothesis (1) and (3), it is possible to test whether the importance of Mid- and South- Atlantic Bights is due to random chance and small sample size. To do so we can either (a) significantly increase the number of females equipped with satellite tags or (b) investigate the reliability of stable isotope analysis as a tool to infer post-nesting migration of a large number of females to obtain a better representation at the population level.

### Relationship between Loggerhead RBC Isotopic Signatures and Post-nesting Migratory Destinations

The variability we found among individuals in both δ^13^C and δ^15^N allowed us to identify three distinct foraging aggregations. Four gradients from enriched to depleted δ^13^C in marine habitats [18], [69], [70], [71], [72], [73] can explain the variability in δ^13^C we observed: (1) nearshore/offshore, (2) benthic/pelagic, (3) enriched/depleted δ^13^C food webs and (4) low/high latitudes.

We reject the hypothesis that differences in δ^13^C are due to a neritic/oceanic gradient because all the loggerheads we tracked stayed on the continental shelf (within the 200 m isobaths), thus in neritic habitat. Our data did not allow testing the benthic/pelagic gradient because we only have dive profile data for four (of the 14) loggerheads we tracked. Bathymetry is not a good proxy to investigate the benthic/pelagic gradient because individuals may use the water column differently and these differences can only be detected if diving profiles are available. Adult loggerheads are known to feed mostly on benthic invertebrates such as crabs and mollusks [35], [74]. Since all loggerheads resided on the continental shelf and remained within their diving limit (up to 233 m: [75]), we hypothesize the majority of their diet will be made of benthos and, thus, exclude a primary role of the benthic/pelagic gradient in driving the differences in δ^13^C among loggerheads. The benthic/pelagic and the enriched/depleted food web gradients are tightly connected. Benthic organisms will most likely feed on seagrass or algae-based webs that are enriched in δ^13^C compared to pelagic environment based on phytoplankton food webs [76]. The last known gradient that could explain variation in δ^13^C is the latitudinal gradient. Latitudinal differences in δ^13^C are due to temperature, surface water CO_2_ concentrations and differences in plankton biosynthesis or metabolism [77]. The loggerheads we tracked moved across a wide latitudinal range (23°N to 38.6°N) and, therefore, provide an opportunity to test the latitudinal gradient hypothesis. The North-South latitudinal gradient in δ^13^C isotopic values of our satellite-tracked loggerheads, with northern individuals being more depleted in ^13^C, support the conclusion that a latitudinal gradient is the main driver of the variation in δ^13^C we observed. This conclusion agrees with previous studies in several marine taxa (cephalopods [78], penguins [79], North Pacific humpback whales [7], Cory’s shearwater [33], albatrosses [19]).

For nitrogen, northern females were the most enriched, and southern females the most depleted, in ^15^N. The relationship between latitude and δ^15^N was weaker than for δ^13^C, suggesting that other factors may affect loggerhead RBC δ^15^N values. Variation in δ^15^N can be explained in three ways: (1) loggerheads at different latitudes forage at different trophic levels, (2) the differences in RBC δ^15^N are a consequence of primary producers’ baseline shift in nitrogen values associated with prevailing N cycling regimes that are maintained and amplified higher up the food chain and (3) a combination of the two hypotheses. The nitrogen stable isotope ratios of primary producers define the δ^15^N value at the base of the food web and are a function of the δ^15^N values of their nutrient sources (e.g. nitrate, ammonium, N), subsequent biological transformation (e.g. nitrogen fixation, which lowers the δ^15^N values of primary producers, and denitrification, a process that increases values of δ^15^N) and isotopic fractionation [13], [80], [81]. Data available in the literature on plankton δ^15^N support a gradient in the NWA, with δ^15^N values becoming progressively more enriched from the subtropics as we move north along the U.S. coastline (McMahon et al. as cited by [13]) [82]. Loggerheads that migrated south moved to areas dominated by N_2_ fixation, where source nitrogen has a lower isotopic composition [81], [83], while loggerheads moving into the MAB entered a region whose nitrogen budget is mostly driven by denitrification and, thus, it is characterized by high phytoplankton δ^15^N value in surface waters [84].

There also may be some individual variability in foraging preference, as reflected in our data on females using northern feeding areas. Within the northern aggregation, our δ^15^N data show two clusters that may reflect two alternative foraging strategies. One group of females (n = 3) has δ^15^N values ranging from 9.74 to 10.28 ‰ (10.07±0.29‰), while the second group (n = 3) δ^15^N values range from 13.77 to 14‰ (13.87±0.12‰). These values suggest that females of the two clusters forage at different trophic levels. Despite previous paradigms that all turtles are benthic foragers, we suspect that the depleted group has a diet based mostly on jellyfish, while the enriched group forages mostly on benthos (crustacean and mollusks). These conclusions are supported by video footage of loggerheads foraging on sea scallop beds in the Mid-Atlantic (Haas et al. unpublished). Intraspecific variability in foraging preference in adult female loggerheads has been demonstrated using series of scute samples [55]. Alternatively, differences in δ^15^N between the two groups may reflect an anthropogenic effect. Recently McKinney et al. [85] found a gradient in δ^15^N of particulate matter available to primary producers from estuaries (more enriched) to nearshore (average 30 km offshore) to mid-shelf (average 90 km offshore) in six locations at the same latitude (in the Mid Atlantic Bight). Our two groups of northern females also followed this pattern, with the enriched group residing an average of 17 km from shore (range = 10–29 km) and depleted group 71 km (range = 67–76 km) from shore. Thus, both groups may forage at the same trophic level and the differences in δ^15^N may be attributed to agriculture runoff and anthropogenic waste that increase δ^15^N in nearshore compared to mid-shelf ecosystems [85]. We cannot discriminate between these alternative hypotheses (different trophic level vs. anthropogenic effect) with our data, but further investigation using additional elements (oxygen and sulfur), compound specific stable isotope analysis, trace minerals and contaminant levels could be informative.

### Discrimination of Stable Isotope Ratios According to Foraging Areas and Assignment of Untracked Females

Our use of the isotopic patterns identified in the 14 loggerheads equipped with satellite tags to assign putative post-nesting migration destinations of the remaining 57 untracked females allowed us to scale up the information obtained with satellite telemetry, gain a better idea at the population level and begin to understand relative importance of foraging grounds. Telemetry and assignment results were similar and highlighted a similar relative importance of foraging grounds. However, it should be noted that while telemetry results were obtained over the course of several years (2008, n = 2; 2009, n = 6; 2010, n = 6), all the untracked turtles analyzed were sampled in 2010. Therefore, our analysis does not take into consideration remigration interval, which may affect the relative importance of each foraging area on a year-to-year basis.

Several authors [18], [26], [77] have called for studies that integrate satellite telemetry data to ground truth the use of isotopic data as proxies for habitat use and diet. Validation of stable isotope analysis with tracking has recently been done in other migratory species (several sea bird species [86], albatrosses [19], kittiwake [52], Procellariiform species [33], fin whales [87]). With regard to sea turtles, a combination of satellite tracking and stable isotope analysis has been used in juvenile [23], adult male [25] and adult female loggerheads nesting in Japan [20] and Greece [24], and adult leatherbacks [34]. Our study, as well as previous studies in loggerheads, supports the use of stable isotope analysis to infer post-nesting foraging grounds. However, while Zbinden et al. [24] found only δ^15^N to be informative in the Mediterranean, our study in the NW Atlantic, as well as Hatase et al. [20] in the NE Pacific, used both δ^13^C and δ^15^N to assign post-nesting migration destinations. Interestingly, Hatase et al. [20] found differences in δ^13^C and δ^15^N to be caused by a neritic/oceanic gradient, while we found them to be associated with a latitudinal gradient. Therefore, while we support the use of stable isotope analysis *in lieu* of more expensive satellite tags, we emphasize the need to validate the use of isotopic signatures with satellite telemetry on a subsample of individuals because oceanographic processes that affect baseline stable isotope ratios differ among ocean basins and geographical regions and, thus, data interpretations without validation can be misleading.

### Conclusions

The Carr NWR hosts approximately 25% of all the nests laid by the NWA loggerhead Florida Peninsular Recovery Unit, which in turn makes up the greatest majority of the NWA female population. Therefore, to identify key foraging areas used by females nesting at Carr NWR is particularly important for the persistence of the species as a whole. Using a combination of satellite telemetry and stable isotope analysis we not only identified prime foraging areas -whose importance was previously unknown- but also validated the use of stable isotope analysis as a tool to derive post-nesting migration destinations for the most important breeding aggregation of this Recovery Unit. We provided the first documentation that the continental shelf of the Mid- and South Atlantic Bights offer essential foraging areas for a large number (61%) of adult female loggerheads of the NWA Florida Peninsular Recovery Unit. These same areas have been found to be extremely important for loggerheads of the NWA Northern Recovery Unit [47], [59], [60]. Our findings suggest that a large proportion of NWA Florida Peninsular Recovery Unit loggerheads are likely to be found within the USA Economic Exclusive Zone, potentially simplifying strategies for the conservation of the two most numerous Recovery Units of the NWA loggerhead populations. We agree with Hawkes’ conclusion [47] that models integrating loggerhead spatial data (e.g. home range, niche models), anthropogenic threat data (e.g. from commercial fisheries and future plans for offshore oil drilling) and climate change are needed to identify hotspots to prioritize for conservation management.

After validating stable isotope analysis with satellite tracking, we suggest using isotopic signatures to assign turtles to foraging regions to scale up knowledge obtained from a limited number of individuals equipped with satellite tags to sample sizes that are more representative at the population level. Regular monitoring of foraging locations for nesting females will open new opportunities to investigate carry-over effects (*sensu* Norris [88]: any event occurring in one season that influences individual performance in a non-lethal manner in subsequent season) and assess variation in relative importance of foraging grounds that, in turn, may reflect changes in environmental conditions (e.g. food availability) or anthropogenic stress (e.g. differential fishing pressure, pollution).

## Supporting Information

Figure S1 **Displacement from release site plot of loggerheads equipped with satellite tags that followed the northern strategy and migrated between summer and winter foraging areas (turtle a–f).** Phases of migration are represented by rapid changes in displacement distance; summer and winter foraging areas can be seen where displacement values plateau. Note differences in y-axis scale among Figure S1, S2 and S3.
(TIF)

Figure S2 **Displacement from release site plot of loggerheads equipped with satellite tags that resided in eastern central Florida (turtle g–j).**
(TIF)

Figure S3 **Displacement from release site plot of loggerheads equipped with satellite tags that followed the southern strategy and took up year-round residence in southern foraging grounds (turtle k–n).** Phases of migration are represented by rapid changes in displacement distance. Year-round foraging areas can be seen where displacement values plateau.
(TIF)
