# Supplementary figures and images for: Inferring Foraging Areas of Nesting Loggerhead Turtles Using Satellite Telemetry and Stable Isotopes
Source: PLoS One. 2012 Sep 20;7(9):e45335. doi: 10.1371/journal.pone.0045335 (PMC3447946; doi:10.1371/journal.pone.0045335)

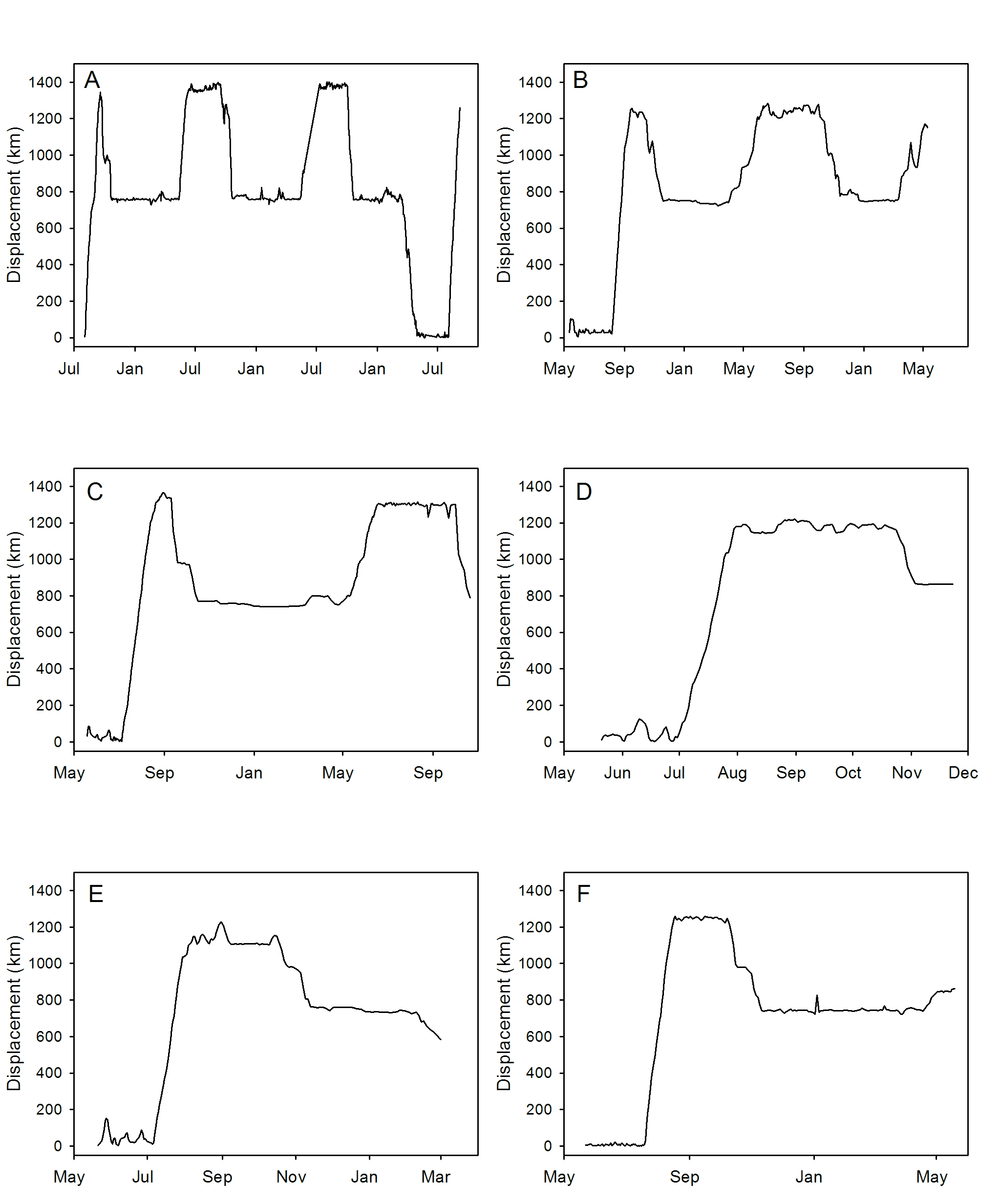

Supplement: Figure S1 — Displacement from release site plot of loggerheads equipped with satellite tags that followed the northern strategy and migrated between summer and winter foraging areas (turtle a–f). Phases of migration are represented by rapid changes in displacement distance; summer and winter foraging areas can be seen where displacement values plateau. Note differences in y-axis scale among Figure S1, S2 and S3. (TIF) [file pone.0045335.s001.tif]

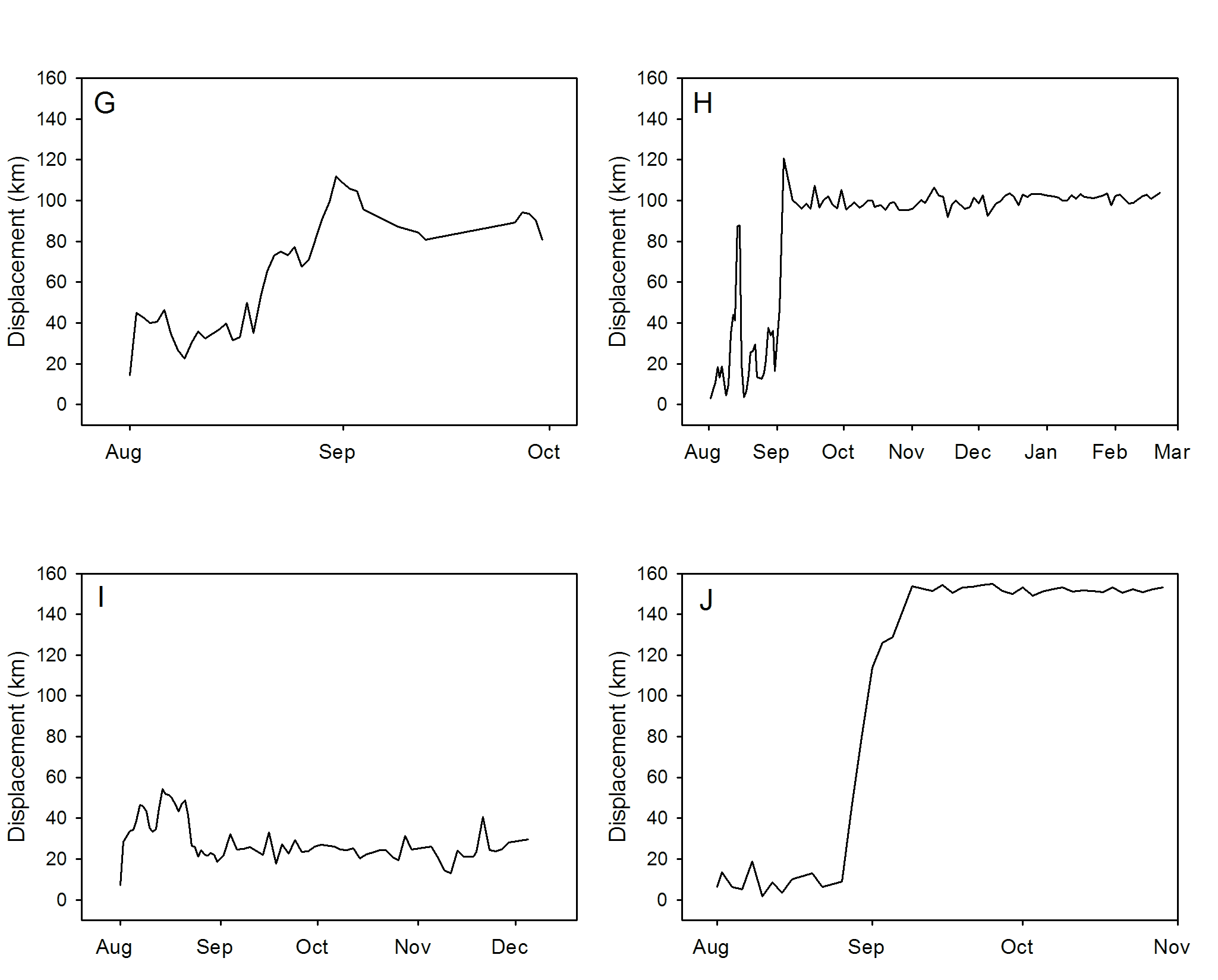

Supplement: Figure S2 — Displacement from release site plot of loggerheads equipped with satellite tags that resided in eastern central Florida (turtle g–j). (TIF) [file pone.0045335.s002.tif]

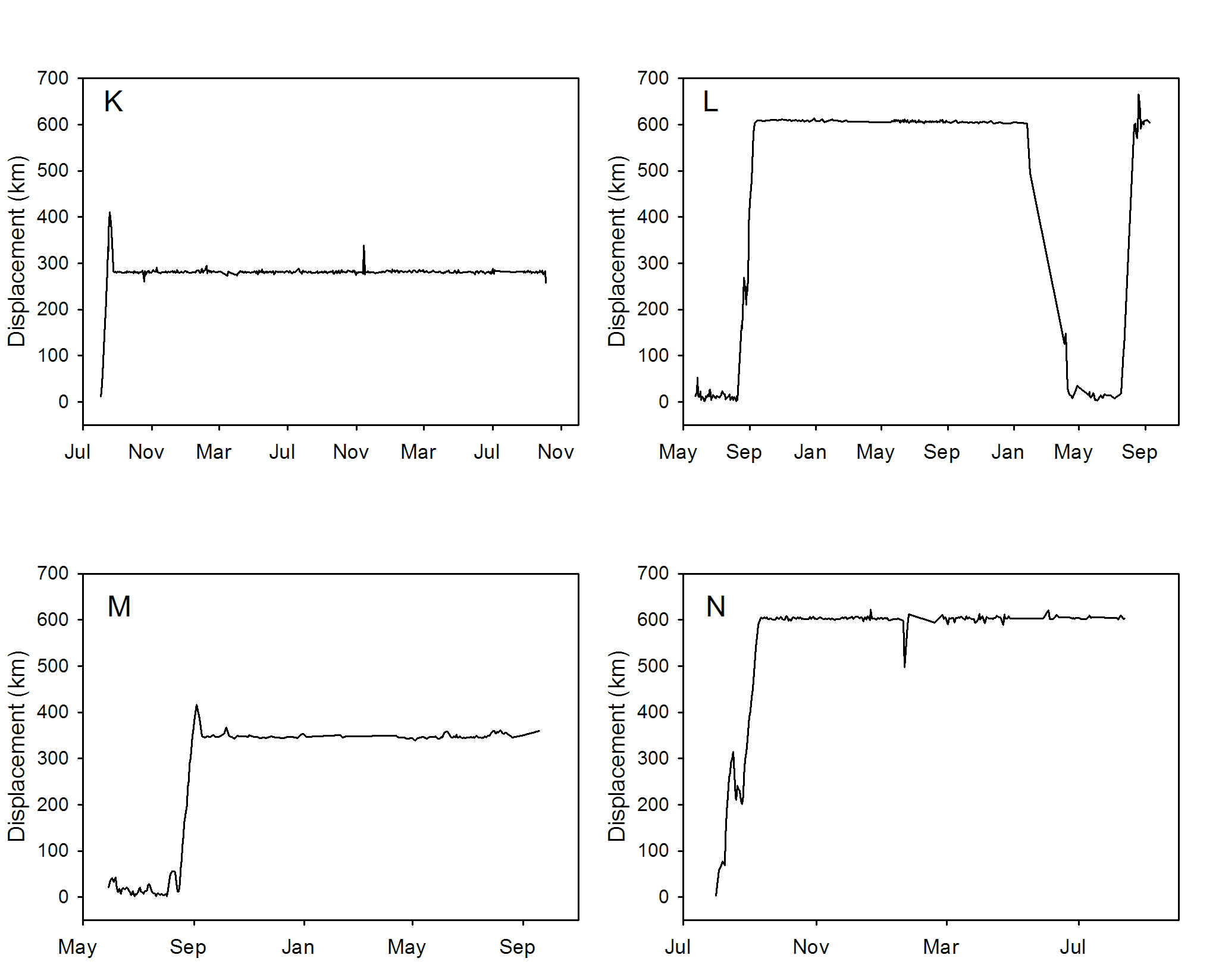

Supplement: Figure S3 — Displacement from release site plot of loggerheads equipped with satellite tags that followed the southern strategy and took up year-round residence in southern foraging grounds (turtle k–n). Phases of migration are represented by rapid changes in displacement distance. Year-round foraging areas can be seen where displacement values plateau. (TIF) [file pone.0045335.s003.tif]
